# Analysis of coastline changes under the impact of human activities during 1985–2020 in Tianjin, China

**DOI:** 10.1371/journal.pone.0289969

**Published:** 2023-11-16

**Authors:** Guangsheng Wang, Zihao Duan, Tong Yu, Zhicheng Shen, Yajing Zhang

**Affiliations:** 1 China Harbor Engineering Company Limited, Beijing, China; 2 National Engineering Laboratory for Port Hydraulic Construction Technology, Tianjin Research Institute for Water Transport Engineering, Tianjin, China; 3 State Environmental Protection Key Laboratory of Simulation and Control of Groundwater Pollution, Chinese Research Academy of Environmental Sciences, Beijing, China; Van Lang University: Truong Dai hoc Van Lang, VIET NAM

## Abstract

The accurate evaluation of shoreline movement is a crucial aspect for managing highly dynamic coasts. This study employed Landsat TM and OLI data through the Digital Shoreline Analysis System model to quantify changes in the spatiotemporal distribution of Tianjin’s coastline from 1985 to 2020. The results showed that the coastline length (CL) increased by 178 km and 151% over the past 36 years, with an average increase of 5.1 km/a. Accretion and erosion processes along the entire coast were observed at rates of 83.9% and 16.1%, respectively. Notably, the Tianjin Port Area and Nangang Industrial Zone showed remarkable changes in the shoreline in 2009. Night lights (NL) were used to display the intensity of human activity in this area, and the spatial heterogeneity of night light intensity was significant. Compared to the total night light (TNL) in 1985, it increased by 116% in 2020. The relationship between TNL and CL was then established and displayed a significant positive correlation (*r* = 0.91). With the increasing total night light, the growth of the CL presented changes with an initial slow increase, then rapid increase, and finally slow increase. In the second phase of TNL, the CL experienced a considerable increase due to anthropogenic activities such as land reclamation and port construction, fueled primarily by government policies during the period of 2005–2013. Subsequently, there was little change in the coastline. These findings provide valuable insights into spatiotemporal coastline monitoring programs and sustainable coastal management.

## 1. Introduction

Information regarding the dynamic changes of the coastline is essential for geographical exploration, coastal erosion or accretion monitoring, and resource management [1]. Approximately 40% of the world’s population lives within 100 km of the seashore, and the increasing demand for land resources has led to extensive reclamation activities (e.g., aquaculture and port terminals) [2]. Consequently, intense human activities and rapid economic development have accelerated the utilization of coastal areas. Therefore, although land reclamation brought great benefits to society, it subsequently also resulted in serious environmental problems such as water quality deterioration and the reduction of marine biodiversity [3]. These activities can have significant effects on coastal ecosystems, including changes in sediment transport, erosion rates, and shoreline morphology, which subsequently affects marine habitats, biodiversity, and ecosystem services [4]. Human activity is one of the main factors driving these changes. Therefore, effective coastal resource management requires thorough monitoring and assessment of shoreline dynamic changes under human activities. In addition, the ability to develop and implement adaptive strategies which can mitigate negative impacts and promote the sustainable use of coastal resources is required.

Understanding the position of the coastline is essential for addressing coastal restoration and erosion control problems. Border detection between land and water bodies is the basis for analyzing coastline position changes. However, utilizing typical ground survey techniques is time-intensive, and it is often difficult to cover a vast area [5]. Recently remote sensing provides an effective tool for evaluating coastline changes due to anthropogenic activities [6, 7]. Kuleli et al. [1] analyzed the shoreline change rate of the Ramsar wetlands in Turkey using Landsat images, and the remarkable shoreline changes were observed in the Yumurtalik and Goksu. Li et al. [8] investigated and quantified the correlations between the level of human activity and shoreline changes using multitemporal Landsat imagery in Xiangshan Bay, China, and Tampa Bay, USA. They found that the intensity of coastal artificialization has had a close correlation with the sinuosity of coastlines in a certain period. Using example-based feature extraction and visual interpretation, Hu et al. [9] investigated shoreline changes in the Greater Bay Area. The result showed that the coastline changes were mainly connected to human activities. These studies mainly focus on the changes in coastline length, land type, and fractal dimension which provide inspiration for research on coastline dynamic monitoring in different regions.

Several studies have been conducted on the coastal area of Tianjin. Based on multitemporal remote-sensing data, Zhu et al. [10] extracted the Bohai Sea coastline and investigated the relationship between shoreline changes and anthropogenic activity. Fu et al. [11] investigated changes in the Bohai Sea coastline and sea area over the last 30 years using Landsat images and quantified the overall changes in coastline length and sea area. Chen et al. [3] analyzed the impact of land reclamation in the Tianjin Binhai New Area on GDP growth and seawater quality. The results revealed that sea reclamation played a vital role in promoting economic growth, but it also caused great damage to the eco-environment. Zhu et al. [12] investigated the changes in shoreline types and harbor reclamation processes in Bohai Bay. The results indicate that the Bohai Sea area shrank by 3% from 2002 to 2018. While Tianjin’s manmade shoreline length expanded by 46.6 km, and the natural shoreline shrank by 47.5 km.

The accurate depiction and quantification of spatial arrangements, in addition to the provision of references for sea area reclamation management, are crucial concerns in the realm of coastal zone management. However, previous research on the quantitative analysis of temporal and spatial shoreline changes in Tianjin and their correlation with anthropogenic activities remains limited and inadequate. In view of this insufficiency, this study endeavors to fulfill two principal objectives: (1) to explore the temporal and spatial variations of shorelines and accretion and erosion areas, (2) to identify the relationship between coastline changes and human activities. Overall, this study expands upon existing research by furnishing a current and comprehensive comprehension of the nature of coastal adjustments in Tianjin, as well as enriching our knowledge of the intricate interactions between human actions and natural processes in the coastal zone.

## 2. Materials and methods

### 2.1. Study area

Tianjin is located along the northeastern area of China, west of the Bohai Sea Bay. The coastline crosses the Binhai New Area (BNA) of Tianjin, starting from the right bank of Jianhekou in the north, ending at the Beipai River in the south. The length of the coastline is 295 km. The land area of the BNA is approximately 2340 km^2^ and the sea area is 3000 km^2^. The BNA covers three urban areas (Hangu, Tanggu, and Dagang districts) [13]. Tianjin Port is the core of BNA and it is the largest artificial port in mainland China. Tianjin’s coastline is a cumulative plain coast, which is typically silty and muddy [14]. The Haihe River is one of the three main estuaries. The coastal areas are mainly divided into six functional zones: the Northern Xinjiang Power Plant (NXPP), Central Fishing Port Area (CFPA), Binhai Leisure Zone (BLZ), Tianjin Port Area (TPA), Port Economic Zone (PEZ), and the Nangang Industrial Zone (NIZ) [15].

### 2.2. Data source

#### 2.2.1. Coastal area images

Since 1972, NASA’s Landsat program has successively launched nine satellites, and has become the longest operating land observation program [16]. Landsat satellite data are an important source for remote sensing research. It has the advantages of good data continuity, moderate resolution, high data quality, and increased time series, which has been widely used in many research fields worldwide [16].

In this study, long-term observation data from Landsat-4/5/7/8 were used to study the dynamic changes in Tianjin’s shoreline. Coastline records were derived from Landsat images over 1985–2020. The temporal resolution of the Landsat remote sensing images was 16 days, and the spatial resolution was 30 m [17]. The Landsat datasets were archived on the Google Earth Engine platform (https://earthengine.google.com/) [18]. Coastline locations were determined using the modified normalized difference water index (MNDWI) [19], which is described in detail in the following section.

#### 2.2.2. Night light images

A strong positive correlation has been observed between nightlight data and human activities, which can reflect the intensity of human activities and socioeconomic parameters [20]. In densely populated areas, social and economic activities are more active, and nighttime brightness values are higher. When the population density was sparse, the brightness value at night was lower. Nightlight images over the period of 1985–2020 were derived from the Prolonged Artificial Nighttime Light Dataset (PANDA). This study was provided by the National Tibetan Plateau Data Center (https://data.tpdc.ac.cn/) [21]. The spatial resolution of the PANDA was 1 km. The nighttime light of the BNA was extracted as an indicator of human activities and analyzed using ArcGIS 10.5.

### 2.3. Methods

#### 2.3.1. The coastline extraction

Remote sensing images have different reflectances for different types of ground objects and thus present different levels of brightness. The normalized difference water index (NDWI) was determined using specific bands through normalized difference processing to highlight water information in images [22]. Based on the NDWI, Xu [23] proposed a modified normalized difference water index (MNDWI) by calculating the ratio of short infrared and green bands. This method significantly improves the discrimination between water bodies and buildings, solves the problem of eliminating shadows in water body extraction, and thus improves accuracy.

Therefore, the MNDWI is widely employed to detect the boundary between land and sea [24–27]. In this study, the MNDWI was used to enhance the difference between wet and dry beach surfaces and extract the coastline. The MNDWI was expressed as follows:

$$MNDWI=\frac{Green-MIR}{Green+MIR}$$ (1)

where Green is the green band, and MIR is the middle-infrared band. The dataset was processed using image correction [5]. This formula was used to highlight the water body, and the threshold was set to achieve image binarization. The dataset was subjected to noise removal, radiometric correction, geometric precision correction, cloud removal, and image stitching processes. Then, the annual coastline derived from Landsat images can be calculated from 1985 to 2020. The details can be found in Li et al. [28].

#### 2.3.2. Intensity of coastline changes

The mean change rate method was employed to characterize the spatiotemporal intensity of CL change [9, 28]. This was used to calculate the change in the CL of Tianjin as follows:

$${LSI}_{mn}=\left(L_{m}-L_{n}\right)/L_{n}(m-n)\times 100\%$$ (2)

where *LSI*_*mn*_ is the intensity of the shoreline length change from year *m* to year *n* (*n*>*m*), *L*_*m*_ and *L*_*n*_ are the shoreline lengths in years *m* and *n*, respectively. The higher |*LSI_mn_*|, the stronger is the intensity of the shoreline change.

#### 2.3.3. Digital Shoreline Analysis System (DSAS) model

The Digital Shoreline Analysis System (DSAS) model is widely applied for estimating the rate of shoreline migration, which was applied to calculate the shoreline changes of Tianjin [29–31]. Coastline changes were determined based on the temporal coastlines and baselines along the transect line. The baseline was established in the landward direction along the general trend of the coastline and was parallel to it. The cross-sectional interval of the transects was 300 m. A linear regression was used to calculate the coastline change rate for each transect. The endpoint rate (EPR), linear regression rate (LRR), and net shoreline movement (NSM) were employed to determine spatial and temporal shoreline changes. The NSM computation provides a time-related variable and determines the rate of change using LRR and EPR. Descriptions of these methods can be found in studies by Moussaid et al. [32] and Himmelstoss et al. [33].

#### 2.3.4. Total night light index

The total night light (TNL) intensity was employed to display the human activities as a light index [34], as follows:

$$TNL=\sum_{i={DN}_{min}}^{{DN}_{max}}\left({DN}_{i}\times n_{i}\right)$$ (3)

where *DN*_*i*_ is the pixel value of level *i* and *DN*_*max*_ and *DN*_*min*_ are the maximum and minimum pixel values, respectively. *N*_*i*_ refers to the number of pixels at level *i* and *n*_*i*_ refers to the total number of pixels.

## 3. Results and discussion

### 3.1. Coastline length changes

**Fig 1:**
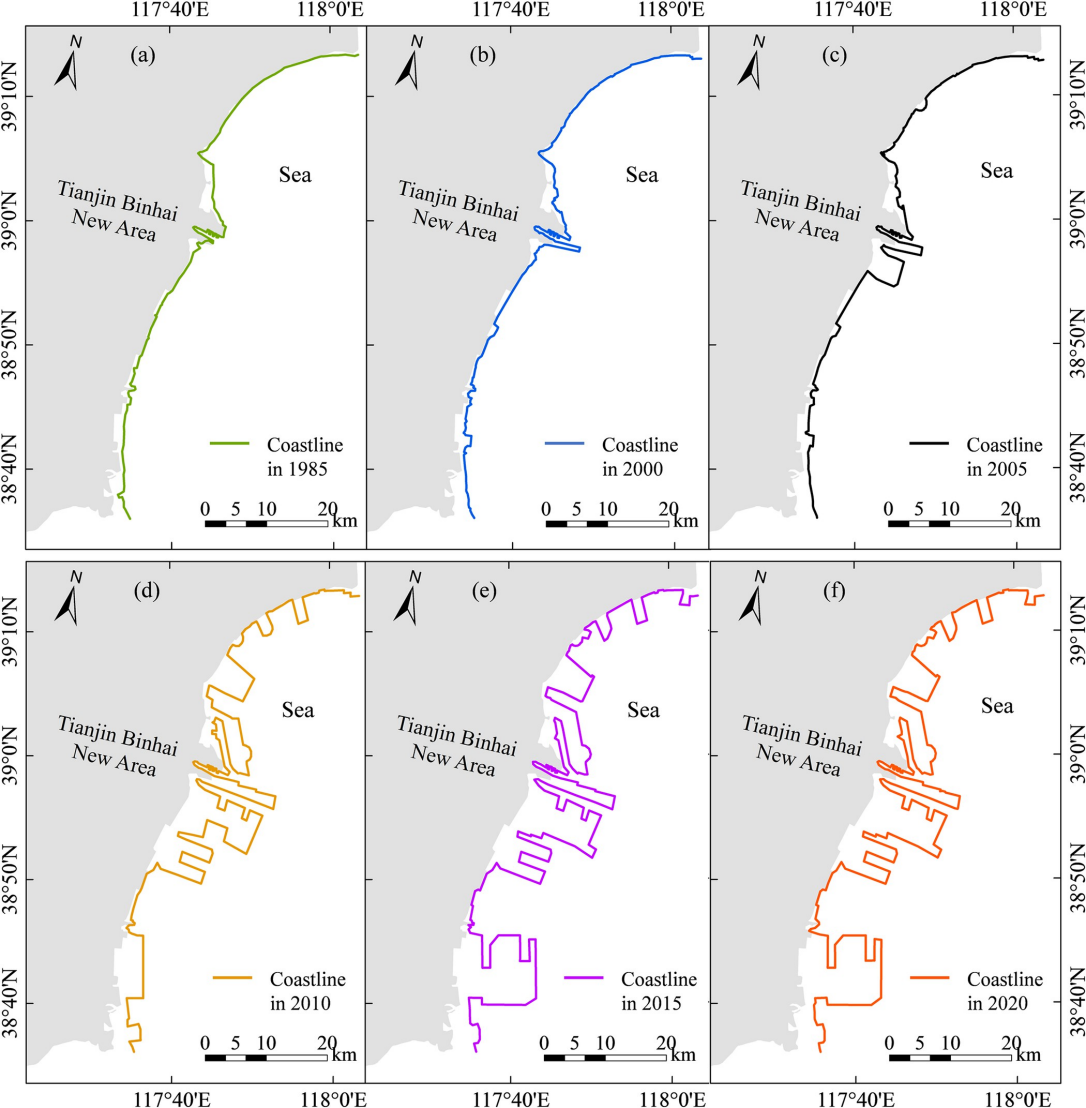
The coastline changes in different years.

**Fig 2:**
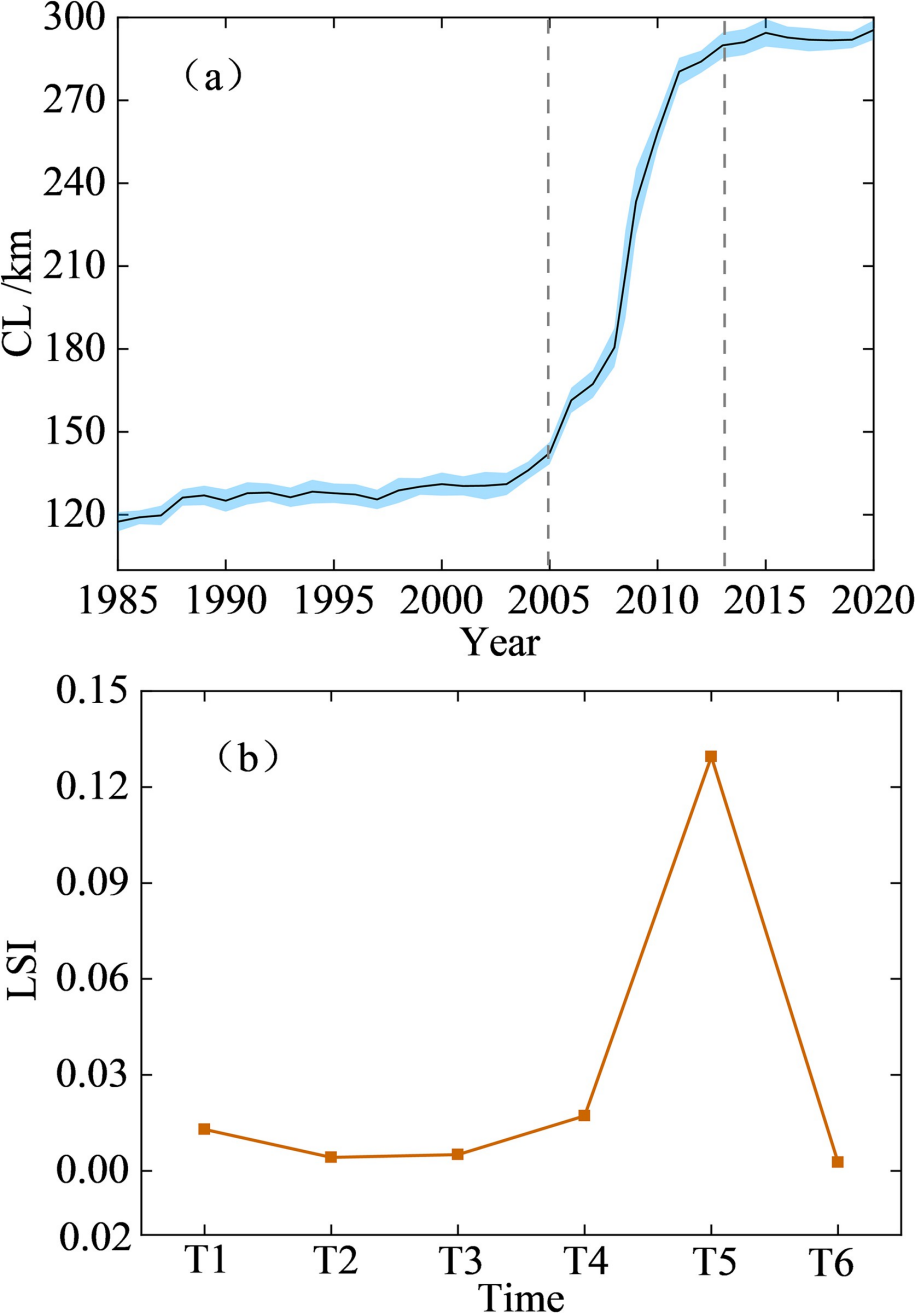
**(a)** The coastline length changes in Tianjin, **(b)** The intensity of coastal length change from 1985 to 2020 (T1 = 1985–1990, T2 = 1990–1995, T3 = 1995–2000, T4 = 2000–2005, T5 = 2005–2013, T6 = 2013–2020).

The coastline length (CL) changes in Tianjin from 1985 to 2020 are shown in Fig 1. The mainland coastline moved towards the sea. The CL increased by 178 km and 151% over the past 36 years, with an annual average increase of 5.1 km/a (Fig 2A). Notably, the growth rate of the CL varied significantly in different periods. The coastline displayed a low rate of increase at 0.8 km/a over the period of 1985–2005. However, the coastline displayed a high increase rate of 17.3 km/a over the period of 2005–2013. The coastline then showed a lower rate of increase, 0.4 km/a, from 2013–2020. The increase in the coastline was primarily due to artificial infrastructure such as reclamation aquaculture, port terminals, and port industrial zones. Coastlines intruded toward the sea area continuously, which lengthen the total coastline.

The intensity of the coastline length index (LSI) varied during different periods (Fig 2B). And the values were displayed in the Table 1. Before T4, the changes in the LSI were steady, and the maximum change value is 0.009. It then displayed an ascending trend from T4 to T5, with a growth of 0.113. It reached the peak value of 0.13 during the period of 2005–2013 (T5). Subsequently, a decreasing trend was then observed from T5 to T6, with a decrease of 0.127. The T5 was considered as the turning point. During the period of 2013–2020 (T6), the proportions of progressive and regressive coastlines were balanced. During the other periods, the progressive coastline was much longer than the regressive coastline.

**Table 1:** The intensity of coastal length change from 1985 to 2020.

	T1	T2	T3	T4	T5	T6
**LSI**	0.013	0.004	0.005	0.017	0.130	0.003

One of the main driving factors leading to this rapid growth of the CL was the increasing intensity of anthropogenic activities during the period of 2005–2013. This is clearly associated with government policies [15]. In 2005, the BNA was upgraded to a third new national-level area and attracted considerable investment in economic development and construction. The continuous expansion of land reclamation towards the sea, as well as the construction of aquaculture areas and ports increased the curvature of the coastline, resulting in an increase in the total CL. Leap frog growth was also observed during this period. In addition, the exploitation of coastline resulted in the dramatic growth in the length of artificial coastline (e.g., aquaculture ponds and harbors), whereas natural coastlines (e.g., sandy and muddy coastlines) also decreased [14].

### 3.2. Accretion and erosion

**Fig 3:**
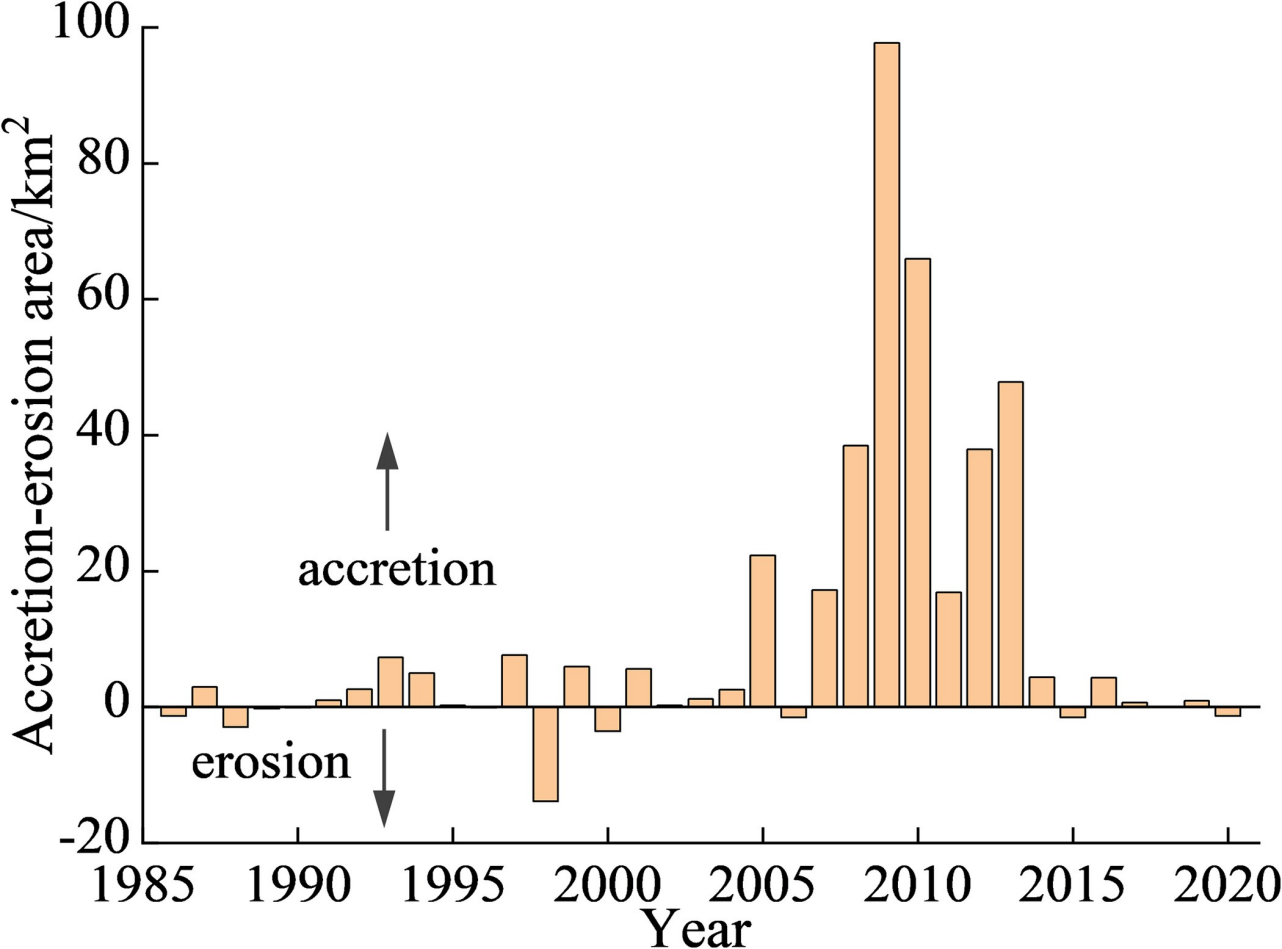
The coastal area changes during the period of 1985–2020.

Variations in the CL led to obvious changes in the spatial extent of the coast. In the early stages, a large number of saltpans and aquaculture ponds were developed along the coast, which reduced the area of tidelands. Subsequently, coastal reclamation construction was mainly undertaken along the coast, which changed the coastline shape and significantly reduced the tideland area. Compared to 1985, the coastline moved seaward 370 km^2^ in 2020 (Fig 3). The progressive and regressive coastlines in the study area exhibited distinct stages. From 1985 to 2004, the coastal areas remained relatively stable with predominant accretion, resulting in a cumulative accretion area of 19.8 km^2^.There was a significant amount of accretion during the period of 2005–2013, and the annual accretion growth reached a peak value of 98 km^2^ in 2009. The Haihe River, whose estuary is located at Tianjin Port, is an important river in Tianjin. However, after the construction of the sluice in 1958, the water and sediment entering the sea decreased sharply, reducing the contribution of sediment deposition along the coastline [35]. These accretions were closely related to the increasing intensity of human activity. By reclaiming the tidelands and sea, a new manmade coastal area was developed, which also contributed to an increase in the CL. Erosion occurred occasionally during some years, with maximum erosion reaching 14 km^2^ in 1998.

### 3.3. Change of temporal coastline

**Fig 4:**
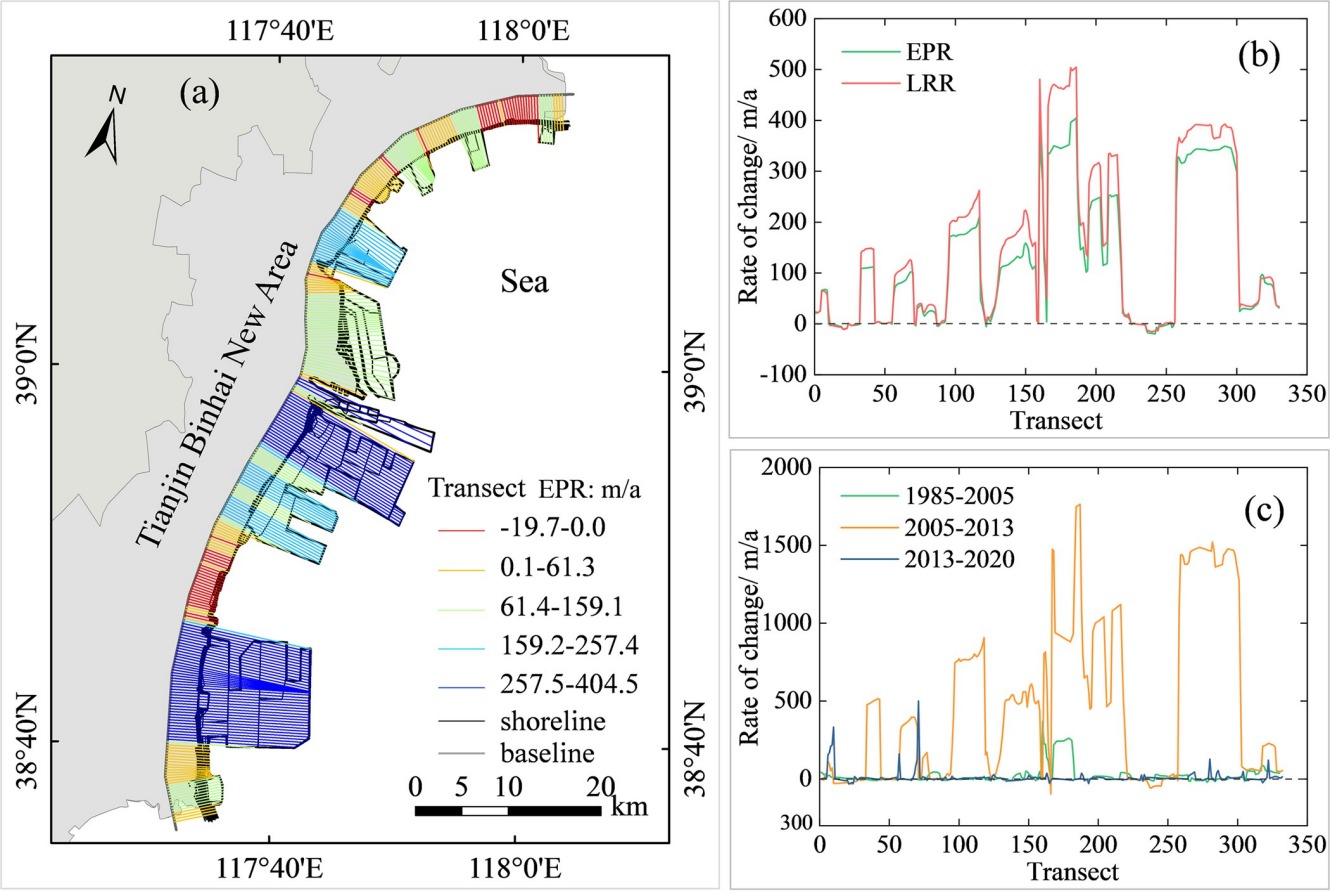
The change rates calculated at each transect along the shoreline from 1985 to 2020 (**(a)** coastline changes based on cast transects; **(b)** coastline change rate based on EPR and LRR; **(c)** coastline change rate during three periods based on EPR).

The coastline change rate was determined using DSAS and three statistical approaches (LRR, EPR, and NSM). Fig 4A shows the transects generated by DSAS, which are perpendicular to the baselines and cross all coastlines. The coastal stretch was classified into five groups based on the shoreline change rates, as measured by EPR. The EPR and LRR values along different transects were calculated, as shown in Fig 4B. The results indicated that 83.9% of the shorelines were accreted. The most significant accretions of 405 and 349 m/a were observed for the TPA (Transect 186) and NIZ (Transect 292), respectively. High erosion rates of 20 and 10 m/a mainly occurred near the NXPP(Transect 242) and PEZ (Transect 21), respectively.

The coastline change rate of Tianjin based on the EPR over three periods, namely 1985–2005, 2005–2013 and 2013–2020, is shown in Fig 4C. The rates of change over these two periods of 1985–2005 and 2013–2020 were relative small. The most significant change rate occurred from to 2005–2013. The average coastline flow rate was 491 m/a. A high accretion of 1764 m/a occurred in the TPA (Transect 187), and a high erosion of 97 m/a occurred near the estuary of the Haihe River (Transect 166). This revealed that the significant enhancement of shoreline changes was caused by human activities, such as land reclamation and aquaculture. From 2013 to 2020, the average coastline rate was 13m/a and was overall relative steady. A high accretion of 501m/a appeared near the CFPA (transect 71), and a high erosion of 31 m/a occurred near the NXPP (transect 21).

**Fig 5:**
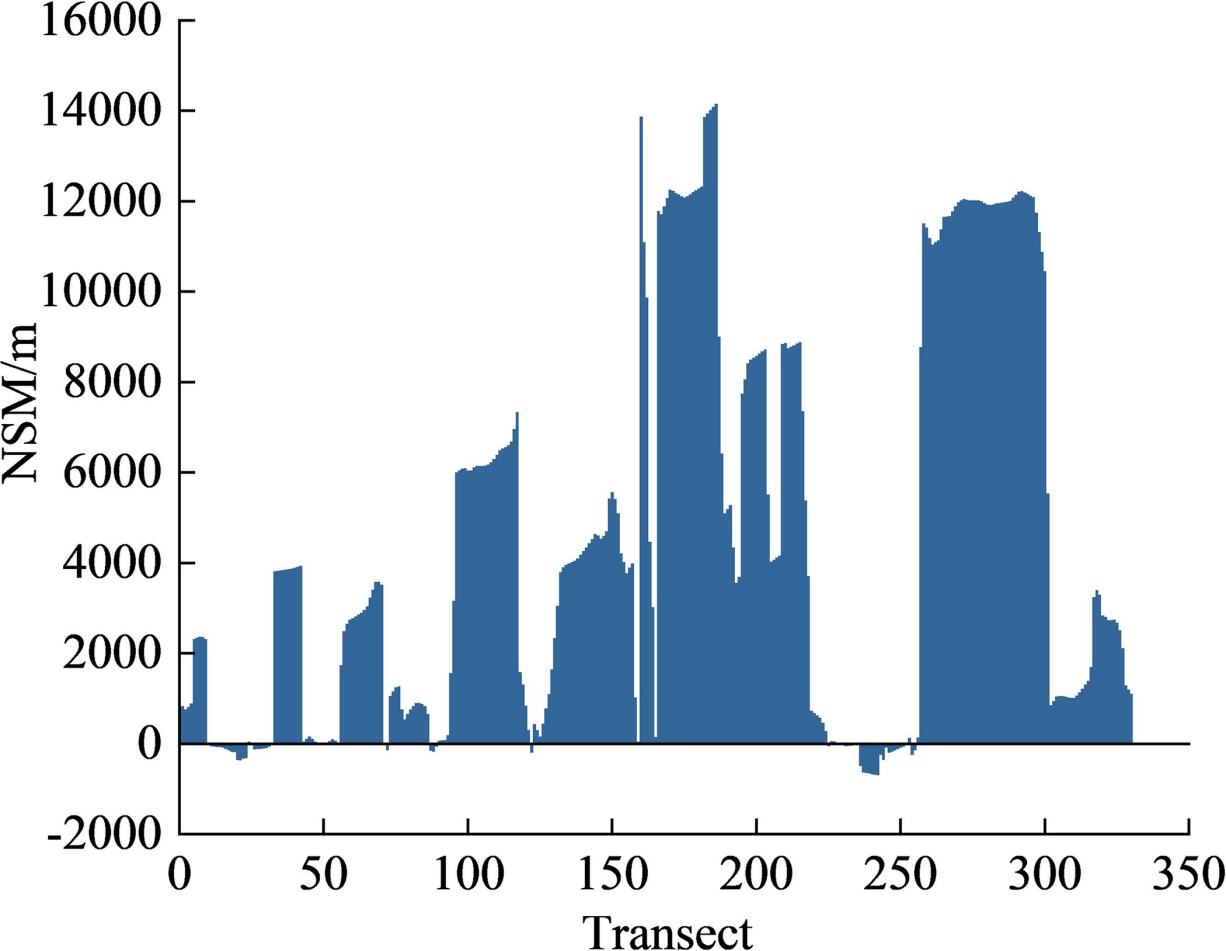
The net shoreline movement (NSM) changes with different transects.

A positive value of NSM indicates that the coastline showed accretion towards the sea and vice versa. As a result, coastlines moved towards the sea by 127.8 m/a during the period of 1985–2020, where 16.1% of the shoreline was under landward erosion (Fig 5). The largest NSM of the coastlines was 14157.6 m, and most of the coastline movement occurred near the NIZ and PEZ. The largest landward NSM was measured at 688.6 m, and it occurred near the seaside resort located between the NIZ and the PEZ. Overall, the coastal areas that experienced significant NSM variations over the period to 1985–2020 were mainly located in the NIZ, PEZ, TPA, and BLZ, whereas the other coastal areas experienced less change. The main reason for these changes was due to the increased human demand for resources with socioeconomic development during that period.

### 3.4. Anthropogenic activities

**Fig 6:**
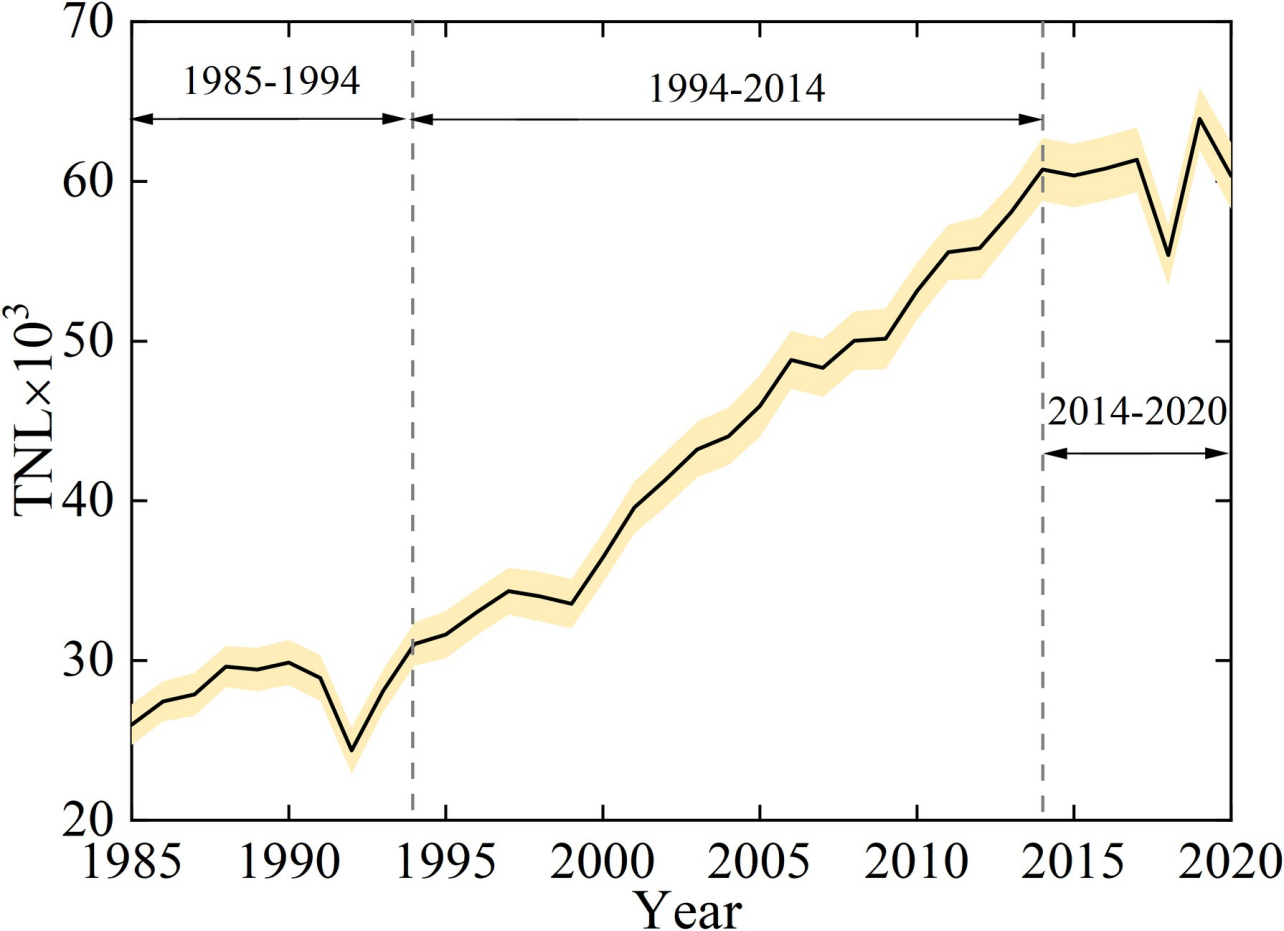
The total night light changes from 1985 to 2020.

Nightlights were found to be a significant explanatory variable for the intensity of human activities [36]. The annual total night light (TNL) was calculated from 1985 to 2020 (Fig 6). The detailed data can be found in the S1 Table. In general, the TNL showed an average annual increase rate of 902. Compared to the TNL in 1985, it increased by 116% in 2020. During the periods of 1985–1994 and 2014–2020, the TNL displayed weak increasing trends which were not significant. The TNL showed a dramatic rise during the period of 1994–2014 from 31000 to 61000. This indicated that the intensity of human disturbances increased significantly during this period, including the expansion of land reclamation and port construction. After the 1994 economic reforms in Tianjin, its economy and competitiveness increased rapidly [15]. Intense anthropogenic activities promoted a rapid increase from 128km to 292km in the coastline length of Tianjin during this period, which can also be seen in Fig 2A. After 2014, the intensity of anthropogenic activities remained relatively steady, and the coastline length exhibited a weak change from 292km to 296km.

**Fig 7:**
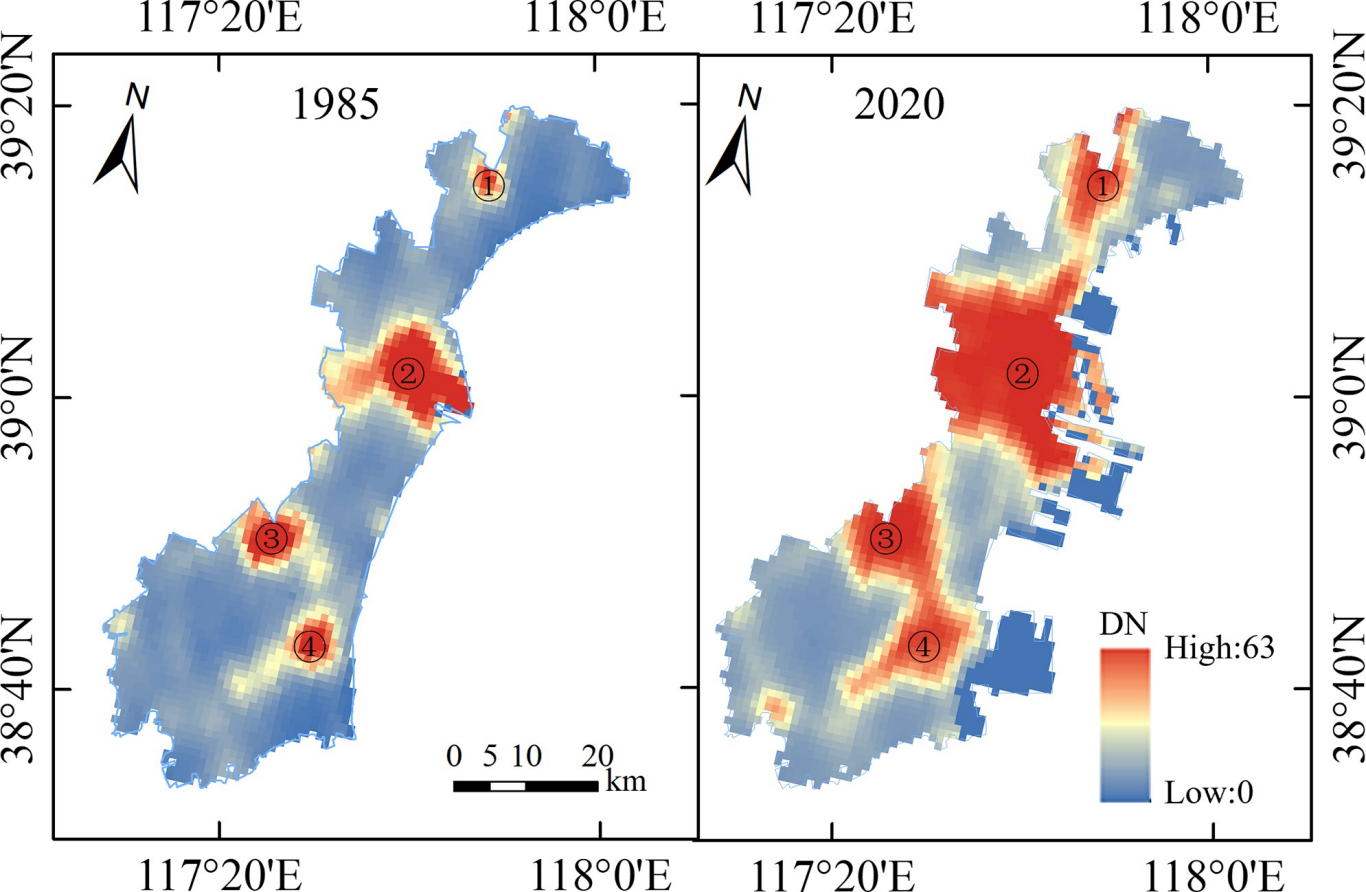
The Spatial distribution of the night light of Binhai New Area in 1985 and 2020 (①Hangu Street Area; ②Center of Tianjin Economic & Technological Development Area (CTEDA); ③Zhongtang Town Area; ④Haibin Street Area).

Meanwhile, to observe the changes of the night light (NL) in different years, the NL spatial distribution of BNA in 1985 and 2020 were shown in Fig 7, respectively. For the timestamp of 1985, the low nighttime lighting region (low or without anthropogenic activities) was dominant, and the high nighttime lighting areas were primarily distributed in four regions: (Hangu Street Area, Center of Tianjin Economic & Technological Development Area (CTEDA), Zhongtang Town Area, and Haibin Street Area). In 2020, a rapid expansion of the highly lit regions (intense anthropogenic activities) was observed near these four regions, especially in the CTEDA. Compared to 1985, the high night light area expanded significantly in 2020. Higher night lights were mainly distributed near the BIZ and TPA.

**Fig 8:**
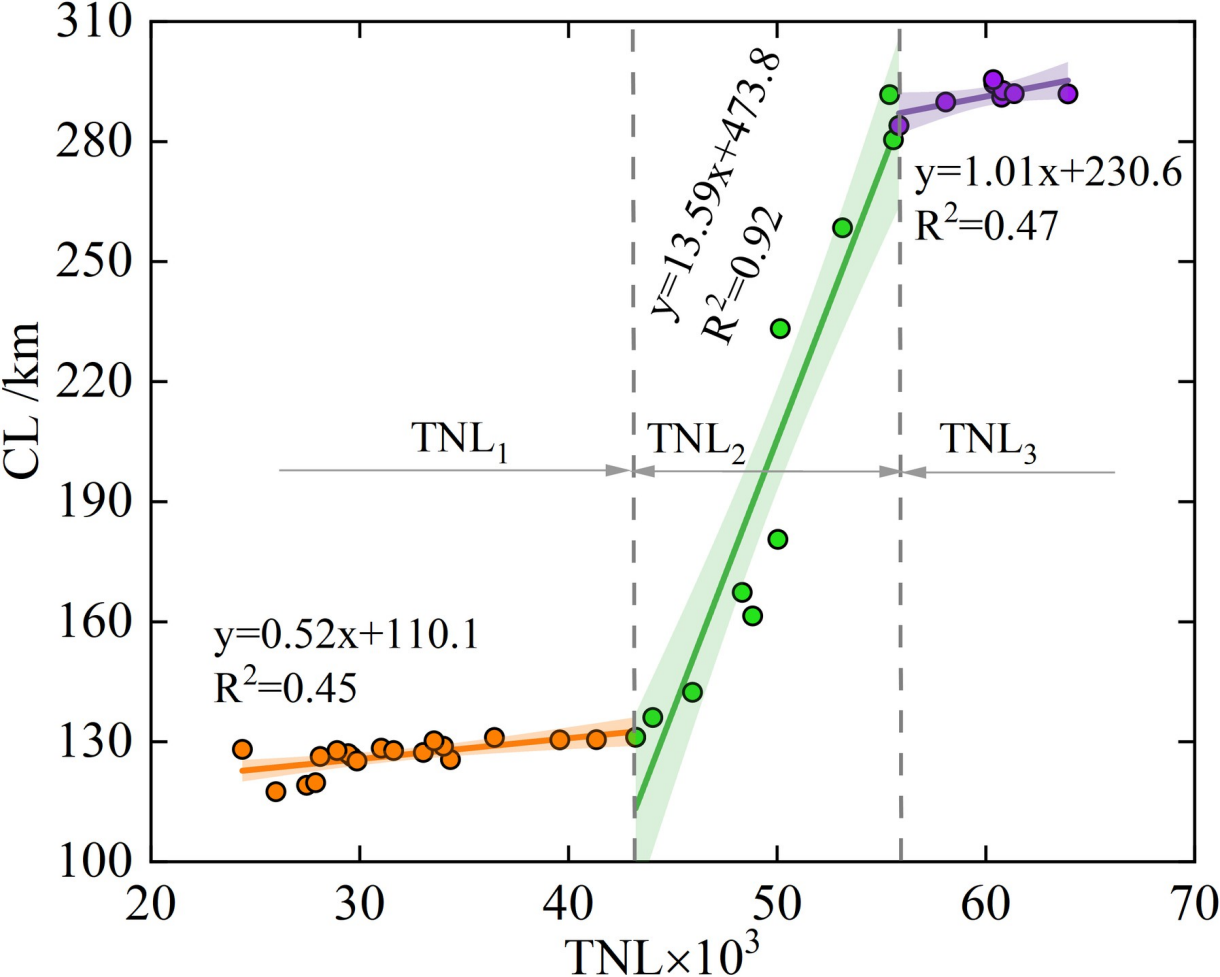
The relationship between coastline length (CL) and the total night light (TNL).

The relationship between the CL and TNL was established (Fig 8). Overall, the CL showed an upward trend with increasing TNL. From the shape of the fitting curve, the spatial growth of the TNL generally presents a "S" shape first of slow growth, then rapid growth, and finally slow growth. The CL changed with increasing TNL in three stages: TNL_1_, TNL_2_ and TNL_3_. Changes in CL were significantly correlated with TNL(r = 0.91), TNL_1_(r = 0.67), and TNL_2_(r = 0.96) (Table 2). In the second phase of TNL_2_, CL increased considerably. Coastline changes were more sensitive to human activities during this phase. Notably, when the TNL increased to a certain extent, CL tended to be stable (TNL_3_), in addition the correlation was not significant. The relationship between the CL and TNL showed that when the total intensity of NL reached a certain level, the TNL could no longer accurately represent the intensity of anthropogenic activities. Therefore, the influence of human-induced impacts on shoreline changes is complex and does not follow a single linear relationship.

**Table 2:** The correlation coefficient between shoreline length and the total night light.

	TNL	TNL_1_	TNL_2_	TNL_3_
**CL**	0.91	0.67	0.96	0.69
**p**	<0.01	<0.01	<0.01	0.06

## 4. Conclusions

Dynamic coastline changes from 1985 to 2020 in Tianjin were investigated, and the probable effects of anthropogenic impacts were assessed. The major conclusions are summarized as follows:

1. The CL increased by 178 km and 151% over the past 36 years, and the mainland moved towards the sea. The LSI changes showed that the intensity of the coastal length change presented a peak value during the 2005–2013. In 2009, a significant amount of accretion occurred in 2009 with a value of 98 km^2^. Human activities, such as reclamation and port construction, were responsible for coastline changes during this period.
2. Through the DSAS analysis, 83.9% of the shorelines showed accretion. The most significant accretions of 405 and 349 m/a were observed in the Tianjin Port Area and Nangang Industrial Zone, respectively. The coastal areas that experienced significant NSM changes during the period of 1985–2020 were mainly located in the Nangang Industrial Zone, Port Economic Zone, Tianjin Port Area, Binhai Leisure Zone, in addition other coastal areas experienced less changes.
3. Correlation analysis revealed that CL was positively correlated with TNL intensity. Compared with the TNL of 1985, it increased by 116% in 2020, and the TNL showed a dramatic rise during the period of 1994–2014. In particular, at the stage of TNL_2_, the CL change was more sensitive to human activity. Anthropogenic factors were considered as the main causes of coastline changes. This was mainly driven by government policies. However, the total intensity of NL reached a certain level, and the intensity of the TNL could no longer accurately represent the intensity of human activities.

## Supporting information

S1 Table The total night light changes from 1985 to 2020. The annual total night light (TNL) was calculated from 1985 to 2020. In densely populated areas, social and economic activities are more active, and nighttime brightness values are higher. When the population density was sparse, the brightness value at night was lower. Nightlight images over the period of 1985–2020 were derived from the Prolonged Artificial Nighttime Light Dataset (PANDA). The spatial resolution of the PANDA was 1 km.
(PDF)

## References

[1] Kuleli T, Guneroglu A, Karsli F, Dihkan M. Automatic detection of shoreline change on coastal Ramsar wetlands of Turkey. Ocean Eng. 2011;38: 1141–1149. doi: 10.1016/j.oceaneng.2011.05.006

[2] Pendleton L, Evans K, Visbeck M. Opinion: We need a global movement to transform ocean science for a better world. Proc Natl Acad Sci U S A. 2020;117: 9652–9655. doi: 10.1073/pnas.2005485117 32295879 PMC7211938

[3] Chen W, Wang D, Huang Y, Chen L, Zhang L, Wei X, et al. Monitoring and analysis of coastal reclamation from 1995–2015 in Tianjin Binhai New Area, China. Sci Rep. 2017;7: 1–12. doi: 10.1038/s41598-017-04155-0 28634414 PMC5478608

[4] Ukhurebor KE, Aigbe UO, Onyancha RB, Ndunagu JN, Osibote OA, Emegha JO, et al. An overview of the emergence and challenges of land reclamation: Issues and prospect. Appl Environ Soil Sci. 2022;2022: 1–14. doi: 10.1155/2022/5889823

[5] Duan Z, Wang X, Sun L. Monitoring and mapping of soil salinity on the exposed seabed of the Aral Sea, Central Asia. Water. 2022;14: 1438. doi: 10.3390/w14091438

[6] Marfai MA, Almohammad H, Dey S, Susanto B, King L. Coastal dynamic and shoreline mapping: Multi-sources spatial data analysis in Semarang Indonesia. Environ Monit Assess. 2008;142: 297–308. doi: 10.1007/s10661-007-9929-2 17874312

[7] Thoai DT, Dang AN, Kim Oanh NT. Analysis of coastline change in relation to meteorological conditions and human activities in Ca mau cape, Viet Nam. Ocean Coast Manag. 2019;171: 56–65. doi: 10.1016/j.ocecoaman.2019.01.007

[8] Li J, Liu Y, Pu R, Yuan Q, Shi X, Guo Q, et al. Coastline and landscape changes in bay areas caused by human activities: A comparative analysis of Xiangshan Bay, China and Tampa Bay, USA. J Geogr Sci. 2018;28: 1127–1151. doi: 10.1007/s11442-018-1546-1

[9] Hu R, Yao L, Yu J, Chen P, Wang D. Remote sensing of the coastline variation of the Guangdong–HongKong–Macao Greater Bay area in the past four decades. J Mar Sci Eng. 2021;9: 1318. doi: 10.3390/jmse9121318

[10] Zhu L, Wu J, Xu Z, Xu Y, Lin J, Hu R. Coastline movement and change along the Bohai Sea from 1987 to 2012. J Appl Remote Sens. 2014;8: 083585. doi: 10.1117/1.JRS.8.083585

[11] Fu Y, Guo Q, Wu X, Fang H, Pan Y. Analysis and prediction of changes in coastline morphology in the Bohai Sea, China, using remote sensing. Sustainability. 2017;9: 900. doi: 10.3390/su9060900

[12] Zhu G, Xie Z, Xu H, Liang M, Cheng J, Gao Y, et al. Land reclamation pattern and environmental regulation guidelines for port clusters in the Bohai Sea, China. PLOS ONE. 2021;16: e0259516. doi: 10.1371/journal.pone.0259516 34731226 PMC8565771

[13] Wang W, Wang YP, Kintrea KK. The (Re)Making of Polycentricity in China’s Planning Discourse: The Case of Tianjin. Int J Urban Reg Res. 2020;44: 857–875. doi: 10.1111/1468-2427.12876

[14] Wang F, Li JF, Shi PX, Shang ZW, Li Y, Wang H. The impact of sea-level rise on the coast of Tianjin-Hebei, China. China Geol. 2019;2: 26–39. doi: 10.31035/cg2018061

[15] Liu Z, Zhang J, Golubchikov O. Edge-urbanization: Land policy, development zones, and urban expansion in Tianjin. Sustainability. 2019;11: 2538. doi: 10.3390/su11092538

[16] Wulder MA, Roy DP, Radeloff VC, Loveland TR, Anderson MC, Johnson DM, et al. Fifty years of Landsat science and impacts. Remote Sens Environ. 2022;280: 113195. doi: 10.1016/j.rse.2022.113195

[17] Duan Z, Wang X, Shakhimardan S, Sun L, Liu W, Luo Y. Impacts of lake water change on vegetation development in the retreat area of the Aral Sea. J Hydrol. 2022;613: 128416. doi: 10.1016/j.jhydrol.2022.128416

[18] Tamiminia H, Salehi B, Mahdianpari M, Quackenbush L, Adeli S, Brisco B. Google Earth Engine for geo-big data applications: A meta-analysis and systematic review. ISPRS J Photogramm Remote Sen. 2020;164: 152–170. doi: 10.1016/j.isprsjprs.2020.04.001

[19] Geng S, Zhang H, Xie F, Li L, Yang L. Vegetation dynamics under rapid urbanization in the Guangdong–Hong Kong–Macao Greater Bay area urban agglomeration during the past two decades. Remote Sens. 2022;14: 3993. doi: 10.3390/rs14163993

[20] Zhou Y, Dong J, Cui Y, Zhou S, Li Z, Wang X, et al. Rapid surface water expansion due to increasing artificial reservoirs and aquaculture ponds in North China Plain. J Hydrol. 2022;608: 127637. doi: 10.1016/j.jhydrol.2022.127637

[21] Zhang L, Ren Z, Chen B, Gong P, Fu H, Xu B. A prolonged artificial nighttime-light dataset of China (1984–2020). National Tibetan Plateau Data Center; 2021. 10.1038/s41597-024-03223-1 PMC11035565 38649344

[22] McFeeters SK. The use of the Normalized Difference Water Index (NDWI) in the delineation of open water features. Int J Remote Sens. 1996;17: 1425–1432. doi: 10.1080/01431169608948714

[23] Xu H. Modification of normalised difference water index (NDWI) to enhance open water features in remotely sensed imagery. Int J Remote Sens. 2006;27: 3025–3033. doi: 10.1080/01431160600589179

[24] Cui BL, Li XY. Coastline change of the Yellow River estuary and its response to the sediment and runoff (1976–2005). Geomorphology. 2011;127: 32–40. doi: 10.1016/j.geomorph.2010.12.001

[25] Ghosh MK, Kumar L, Roy C. Monitoring the coastline change of Hatiya Island in Bangladesh using remote sensing techniques. ISPRS J Photogramm. 2015;101: 137–144. doi: 10.1016/j.isprsjprs.2014.12.009

[26] Kelly JT. Gontz AM. Using GPS-surveyed intertidal zones to determine the validity of shorelines automatically mapped by Landsat water indices. Int J Appl Earth Obs Geoinf. 2018;65: 92–104.

[27] Feyisa GL, Meilby H, Fensholt R, Proud SR. Automated water Extraction Index: A new technique for surface water mapping using Landsat imagery. Remote Sens Environ. 2014;140: 23–35. doi: 10.1016/j.rse.2013.08.029

[28] Li J, Ye M, Pu R, Liu Y, Guo Q, Feng B, et al. Spatiotemporal change patterns of coastlines in Zhejiang Province, China, over the last twenty-five years. Sustainability. 2018;10: 477. doi: 10.3390/su10020477

[29] Frihy O, Deabes E. Erosion chain reaction at El Alamein Resorts on the western Mediterranean coast of Egypt. Coast Eng. 2012;69: 12–18.

[30] Ford M. Shoreline changes interpreted from multi-temporal aerial photographs and high resolution satellite images: Wotje Atoll, Marshall Islands. Remote Sens Environ. 2013;135: 130–140. doi: 10.1016/j.rse.2013.03.027

[31] Yan D, Yao X, Li J, Qi L, Luan Z. Shoreline change detection and forecast along the Yancheng coast using a digital shoreline analysis system. Wetlands. 2021;41: 1–16. doi: 10.1007/s13157-021-01444-3

[32] Moussaid J, Fora AA, Zourarah B, Maanan M, Maanan M. Using automatic computation to analyze the rate of shoreline change on the Kenitra coast, Morocco. Ocean Eng. 2015;102: 71–77. doi: 10.1016/j.oceaneng.2015.04.044

[33] Himmelstoss EA, Henderson RE, Kratzmann MG, Farris AS. Digital Shoreline Analysis System (DSAS) Version 5.0 User Guide-File report. Open: United States Geological Survey. Reston, Virginia: United States Geological Survey; 2018–1179.

[34] Levin N, Kyba CCM, Zhang Q, Sánchez de Miguel AS, Román MO, Li X, et al. Remote sensing of night lights: A review and an outlook for the future. Remote Sens Environ. 2020;237: 111443. doi: 10.1016/j.rse.2019.111443

[35] Sun B, Zuo S, Xie H, Li H, Yang Z. Analysis of impact effects and changes of the coastline in the Bohai Bay during the past 40 years. J East China Norm Univ. 2017;4: 139–148.

[36] Ma T, Zhou Y, Zhou C, Haynie S, Pei T, Xu T. Night-time light derived estimation of spatio-temporal characteristics of urbanization dynamics using DMSP/OLS satellite data. Remote Sens Environ. 2015;158: 453–464. doi: 10.1016/j.rse.2014.11.022

