# Supplementary material for: Analysis of coastline changes under the impact of human activities during 1985–2020 in Tianjin, China
Source: PLoS One. 2023 Nov 16;18(11):e0289969. doi: 10.1371/journal.pone.0289969 (PMC10653502; doi:10.1371/journal.pone.0289969)
Supplement: S1 Table — The annual total night light (TNL) was calculated from 1985 to 2020. In densely populated areas, social and economic activities are more active, and nighttime brightness values are higher. When the population density was sparse, the brightness value at night was lower. Nightlight images over the period of 1985–2020 were derived from the Prolonged Artificial Nighttime Light Dataset (PANDA). The spatial resolution of the PANDA was 1 km. (PDF) [file pone.0289969.s001.pdf]

S1 Table. The total night light changes from 1985 to 2020

Year	Total night light index
1985	25986.28
1986	27447.19
1987	27887.32
1988	29618.39
1989	29443.38
1990	29870.47
1991	28911.07
1992	24376.37
1993	28098.02
1994	31030.66
1995	31623.29
1996	33049.22
1997	34342.09
1998	34015.05
1999	33560.38
2000	36454.74
2001	39578
2002	41338.68
2003	43216.16
2004	44040.07
2005	45940.2
2006	48826
2007	48326.01
2008	50025.3
2009	50145.66
2010	53132.06
2011	55563.36
2012	55823.95
2013	58073.02
2014	60744.97
2015	60369.93
2016	60807.42
2017	61351.32
2018	55384.99
2019	63923.3
2020	60355.44
